# Bilateral True Giant Aneurysm of the Profunda Femoral Artery: Case Report and Review of the Literature

**DOI:** 10.1155/2012/730518

**Published:** 2012-08-09

**Authors:** Alessandro Robaldo, Mauro Maselli, Daniele Maggio

**Affiliations:** Vascular and Endovascular Surgery Unit, Cellini Clinic, Via Cellini 2, 10126 Turin, Italy

## Abstract

We report a rare case of a bilateral true giant aneurysm of the profunda femoral artery aneurysms (PFAAs) in a 80-year-old man with a previous history of “open” abdominal aortic surgery and small bilateral popliteal artery aneurysm. In the English Literature only seven previously cases of true bilateral PFAAs are reported. Due to its location, this lesion may require surgical intervention and removal. The presentation, the diagnostic evaluation, and the surgical management of the aneurysm are discussed.

## 1. Introduction

Profunda femoral artery aneurysms (PFAAs) are extremely rare in comparison to pseudoaneurysms of the same artery. Most of the cases are unilateral and occur with synchronous aneurysms elsewhere. The majority of PFAAs are asymptomatic most of the time. In addition to the risk of rupture, they can cause local pressure symptoms with nerve and vein compression and thrombosis in some cases. The identification of an aneurysm in such a rare location requires a careful physical examination and color Doppler ultrasound. Multidetector CT scanning is especially recommended to avoid missing other aneurysms and occlusive arterial lesions [1]. Good-risk patients with a PFAA >2 cm should undergo elective repair. We present a case of a man with previous history of abdominal aortic surgery, diagnosed with bilateral PFAA after the appearance of bilateral pulsatile mass in the groins.

## 2. Clinical History

**Figure 1:**
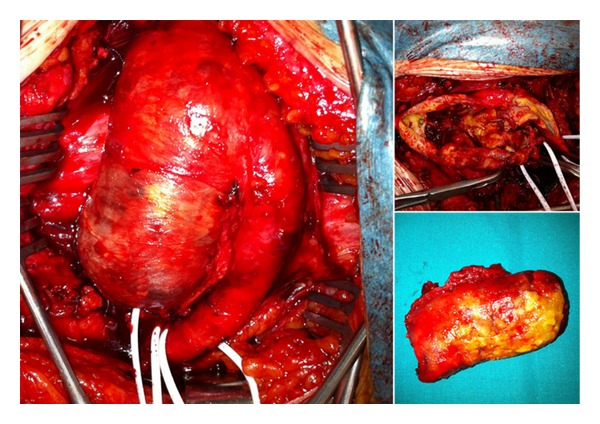
Intraoperative image of the true giant left profunda femoral artery aneurysm. On the right, the aneurysm is opened with a large thrombus.

**Figure 2:**
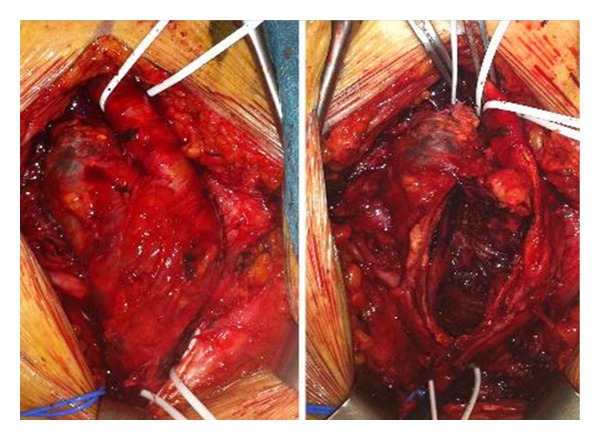
Intraoperative image of the true giant right profunda femoral artery aneurysm.

**Figure 3:**
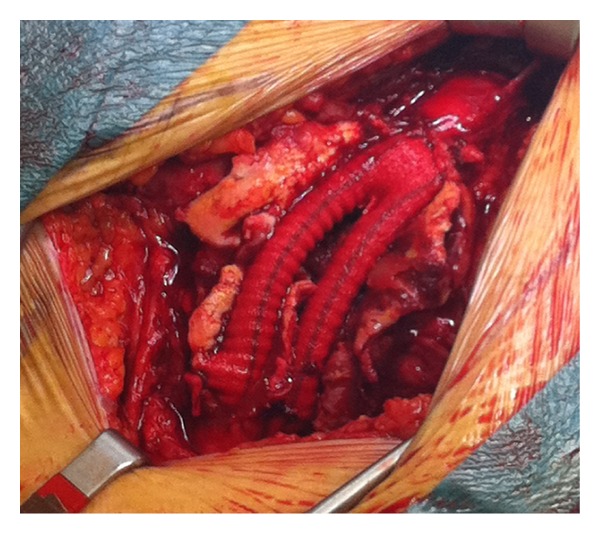
Intraoperative image of the 12 × 6 mm Dacron bifurcated bypass grafting from the proximal common femoral artery to the distal segment of the profunda and superficial femoral artery, respectively.

A 80-year-old male was referred to our attention for a bilateral pulsatile mass in the groin, without any signs of limb-threatening ischemia. The patient had a previous “open” surgery for an abdominal aortic aneurysm in another hospital. History of cardiac and major pulmonary surgery was recorded. Risk factors included hypertension, chronic renal insufficiency, dyslipidemia, and chronic venous insufficiency. No antecedent trauma and local/systemic infection were reported. An ultrasound scan of both groins indicated a bilateral profunda femoral artery aneurysm (PFAA) involving the distal common femoral artery (right maximum diameter: 50 mm; left: 45 mm). The superficial femoral artery was neither occluded nor dilated bilaterally. (Figures 1 and 2). We found a bilateral small aneurysm of the popliteal artery (right: 16 mm; left: 15 mm). Under spinal anesthesia, the patient underwent repair of PFAA through a staged bilateral groin surgical approach. The PFAA was opened, and a large thrombus was removed. Therefore, we replaced the femoral bifurcation performing a 12 × 6 mm Dacron bifurcated bypass grafting with a 5–0 prolene continuous suture from the proximal common femoral artery to the distal segment of the profunda and superficial femoral artery, respectively (Figure 3). The patient had an uneventful postoperative course. The patient was discharged in good general condition with regular pulse. At 3-month followup, neither foot nor digital ischemia, complain of paresthesias, pain, discomfort or walking limitation has been observed. Duplex scan showed a good patency in absence of stenosis, pseudoaneurysms, or recurrent aneurysms.

## 3. Comments

The majority of the PFAAs are rare and usually secondary to trauma, infection, or iatrogenic injury. In the English Literature only seven previously cases of true bilateral PFAAs are reported [2]. Patients with PFAA have other aneurysms (abdominal aortic aneurysm, aortoiliac aneurysm, common femoral aneurysm, and popliteal aneurysm) identified in 45–81% of cases [3]. The diagnosis of PFAA can be difficult because of their rarity. Ultrasound scanning is recommended to establish the diagnosis. In our case, the patient had a preoperative workup for an abdominal aortic aneurysm open repair, but the PFAAs were not detected because the scan was taken only to the level of the proximal CFA. The standard of treatment is usually surgical and depends on the blood flow in the femoral-popliteal segment [4]. Indeed, the ligation of PFAA can be an option when patency of the femoral-popliteal segment is observed. However, in our case, we decided to preserve the blood flow to collateral circulation in the lowers limbs, considering the good back-bleeding from the distal profunda femoral artery. We could not use the autogenous vein graft because of the inadequate superficial varicose veins and the size of the nonaneurysmatic segment of the femoral arteries. To the best of our knowledge, the endovascular treatment (transcatheter coil embolization) is described as possible alternative. In the literature only three patients were treated with this technique. It seems to be a safe and minimally invasive alternative to open repair in selected patients in the absence of concomitant disease of the common femoral artery and the origin of the profunda femoral artery [5].

## 4. Conclusion

The bilateral giant true PFAA is a rare entity. The DS exam can provide all the information necessary for the correct management. The lower limbs should be examined carefully when an abdominal aortic aneurysm is found because patients may have synchronous aneurysms in their lower limbs. Treatment should be individualized time by evaluating patients comorbidities and aneurysm conformation.
